# In Search of Covalency Measure of Gd(III)-Ligand Interactions

**DOI:** 10.1021/acs.jpclett.4c01903

**Published:** 2024-09-17

**Authors:** Rafał Janicki, Miłosz Siczek, Przemysław Starynowicz

**Affiliations:** University of Wrocław, Faculty of Chemistry, F. Joliot Curie 14, 50-383 Wrocław, Poland

## Abstract

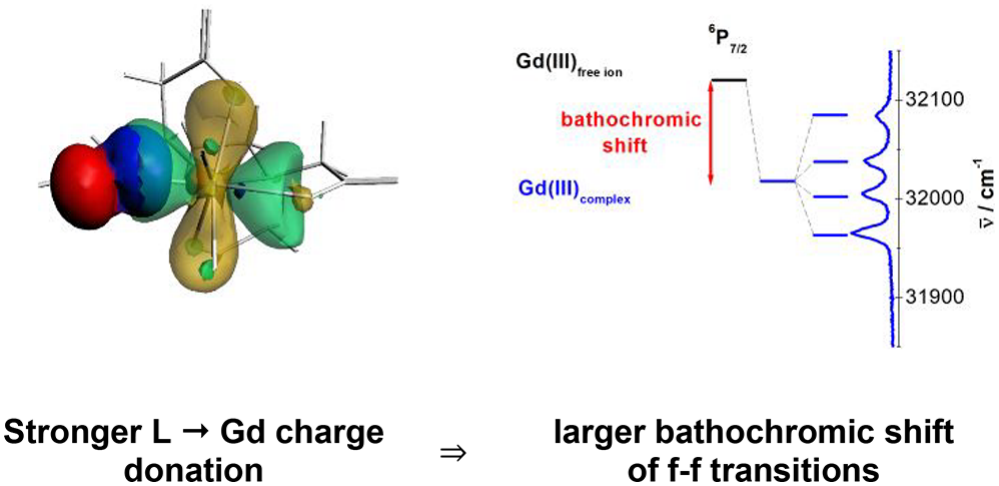

Experimental electron density distribution of the [C(NH_2_)_3_]_3_[Gd(EDTA)F_2_]·H_2_O crystal was determined. The derived experimental and theoretical (DFT) topological parameters such as ∇^2^ρ_c_, ρ_c_, bond degree (BD), kinetics, and potential energy were used to study the nature of Gd–O, Gd–F, and Gd–N interactions. The natural charge of the Gd is 1.86; the natural configuration of the cation is [Xe]6s^0.13^4f^7.10^5d^0.83^, and the covalency of the Gd–L bond is mainly connected with the transfer of charge from the sp^*x*^ ligand orbitals onto the 5d orbitals of the Gd cation. Simultaneously, the donation of charge onto the 6s and 4f orbitals occurs to a lesser extent. Moreover it was found that the donation of the ligand charges onto the Gd(III) is larger for compounds with a lower coordination number. The obtained topological parameters were analyzed in the context of the Gd(III) f–f transition properties, i.e., energy of the excited ^2*S*+1^L_J_ states, Judd–Ofelt intensity parameters, and luminescence lifetimes, of 18 Gd(III) compounds with various O, N, and F donor ligands (DOTA, EDTA, CDTA, DTPA, NTA, EGTA, ODA, F^–^, H_2_O, and CO_3_^2–^). The calculated nephelauxetic β parameter may reflect the penetration degree of electron lone pairs of ligands inside the metal basin. Finally, it was found for the first time that the sum of the Gd(III)–L bond energy (∑*E*_GdL_) is correlated with the position of the gravity center of the ^8^S_7/2_ → ^2*S*+1^L_J_ transitions and increase of covalency of the Gd(III)–L bonds is associated with decrease of their bond energy. The obtained results may shed light on chemical bonding in systems containing f-elements. Such subtle differences in the covalent contribution to the Ln–L or An–L bond may tune the selectivity of the partitioning processes of lanthanides and actinides.

Interactions of the lanthanide(III) cations with ligands are generally considered to be ionic. Many different properties of the lanthanide compounds derived from the electrostatic model are, in principle, consistent with the experimental data. For instance, the stability constants of Ln(III) complexes^1−3^ as well as thermodynamic functions Δ*H*, Δ*S*, and Δ*G* of hydration of Ln(III) aquaions^4^ were calculated from the modified Born’s equations.^5^ Some properties of the solids were also predicted from the point charges model. The Madelung constant derived from X-ray structural data of simple inorganic compounds has been used successfully to determine the lattice energy of crystals.^6^ Furthermore, the ionic model has been employed to predict the crystal field splitting of the ^2*S*+1^L_J_ states in lanthanide and actinide compounds.^7−9^ There is no doubt that the share of the ionicity to the Ln(III)–L interactions is in principle predominant; thus, at a first glance, this kind of interaction may conceal other, less noticeable, covalent interactions. This problem is important not only for a deeper understanding of chemical bonding in f-element systems but also for industrial separation processes of lanthanides for modern technology and actinides for the nuclear industry. Subtle differences in the covalent contribution to the Ln–L and An–L bond may provide and/or enhance selectivity of partitioning processes.^10,11^

From the point of view of the structural criteria, the covalent bond should be directional, as opposed to ionic interactions; moreover, the effective ionic radius of a ligand should be unchanged for ionic compounds. However, the statistical analysis indicates that the Ln(III)–L bond length is the sum of the ionic radii of the Ln(III) cation, the donor atom, and an increment that is a function of the coordination mode (monodentate, bridging, etc.).^12^ The covalent effect is also envisaged in the inverse-trans-influence in tetravalent lanthanide and actinide bis(carbene) complexes,^13^ and it is manifested in the thermodynamic stability of f-element compounds. The calculated standard enthalpy of formation of the actinide (U, Np, and Pu) tribromides and triodides within the mixed ionic–covalent approach follows more precisely the experimental data than those obtained within a purely ionic model.^14^ Jensen and Bond demonstrated that the higher selectivity of organodithiophosphinic acid ligands for the An(III) cations in comparison with the Ln(III) cations does not result from shortened An–S bonds or variations of the structures or composition of the complexes compared to the Ln(III) analogues, but rather it is likely covalence-driven.^15^ As was shown by Siekierski et al., stability of the Ln(III) complexes changes nonmonotonically along the lanthanide and actinide series.^16^ Accordingly, the observed effect, called by them “the double-double effect”, originates in the partially covalent character of the Ln/An–L bond.^17^

Finally the covalence effect may be also manifested in the reactivity of compounds. It has been demonstrated that although the covalent orbital mixing is similar for Ce(C_8_H_8_)_2_ and U(C_8_H_8_)_2_, the differences in orbital overlap results in a greater covalent contribution to the stability of U(C_8_H_8_)_2_, while Ce(C_8_H_8_)_2_ exhibits greater reactivity.^18^ Experimental evidence from Schelter et al. indicates that the Th(IV)-imido fragments in the Th(IV) complexes with (tris(2-*tert*-butylhydroxylaminato)benzylamine) and 3,5-bis(trifluoromethyl)amide anion are more than 3 orders of magnitude more basic than their Ce(IV) isostructural analogues. These findings may indicate that Ce(IV) exhibits a markedly greater degree of covalent character than its early actinide counterparts, such as Th(IV).^19^

Among various physical methods and techniques, spectroscopic methods are the most widely employed to elucidate the nature of the Ln–L/An–L bonds. For example, the position and splitting of the ν_CO_ band in the IR spectra of the lanthanide(III) tetracarbonates may be used as a measure of the coordination number change and the Ln-O(CO_3_^2–^) bond properties. The stretching force constant values (*f*_LnO_), monotonically increase with shortening of the Ln–O(CO_3_^2–^) bond lengths, and this may be attributed to a greater covalence contribution.^20,21^ Binding of a ligand to a Ln–An cation results in perturbation of the electronic structure of the former. This effect manifests itself in bathochromic shift of the n−π* transitions, as was observed in the UV–vis spectra of the N-(methylene-2-pyridine)-N,N-di(methylenephosphonate) complexes with Eu(III) and Tb(III).^22^ Interesting results were obtained from the Mössbauer spectra of ^129^I in solid LaI_3_, GdI_3_, and ErI_3_.^23^ These compounds are characterized by a negative isomer shift, which is similar to that observed in the spectra of CuI. Although the authors stated that iodine ligand in LnI_3_ could be very similar to that in typical ionic compounds (KI, NaI, etc.), there is no doubt that the Cu(I)–I bond is regarded as substantially covalent.^24^ Other experimental results, such as the hyperfine structure observed in the EPR spectra of Ln(III),^25^ the NMR hyperfine coupling in the ^17^O NMR spectra,^26^ or even the vibronic coupling observed in the luminescence spectra,^27^ can also provide useful information about the nature of the Ln(III)–L bond, although the acquired overall picture appears to be only qualitative. A more quantitative analysis of the bond nature may be provided by K-edge X-ray absorption spectroscopy (XAS), as the transition intensity in XAS spectra is directly related to the orbital mixing coefficient.^28,29^ Interesting results were obtained from XAS measurements of the [MCl_6_]^*n*−^ complexes in the solid state; namely, substantial mixing between the Cl 3p and Ln/An 5*d*/6d orbitals was observed, which, however, decreases along the lanthanide series. In addition, the 4f orbitals were found to be only marginally involved in covalent bonding, while 5f (An(IV)) and 3p (Cl) orbital mixing increased for heavier actinides.^30,31^ In general, high-resolution UV–vis–NIR electronic spectroscopy may provide important information regarding the electronic structure of the f-electron ions.^12,32−37^ It is for this reason the UV–vis–NIR spectroscopy was successfully used to find, among other things, the solution stoichiometry and structure of the Gd(III) chelate complexes used as MRI contrast agents.^38^ On the other hand, the f–f transitions observed in the UV–vis–NIR spectra of the lanthanide and actinide compounds may give insight into the nature of the Ln/An-ligand interactions. The theoretical study within ab initio ligand field theory (AILFT) of the series of Na_3_[Ln(ODA)_3_]·2NaClO_4_·6H_2_O crystals (where Ln = Ce–Yb and ODA is the oxidiacetate anion) led the authors to conclude that the inclusion of anisotropic π-bonding is essential to accurately describe the crystal field splitting of the 4f states.^39^ On the other hand, the bathochromic shift of f–f^40−43^ and/or f–d bands^44^ (the nephelauxetic effect, which was observed for the first time by Efraim and coauthors,^45^ and further investigated by, among others, Jørgensen^46^), may also reflect the character of the Ln/An-ligand interaction. Based on this concept, Jørgensen^46^ derived empirical parameters: *h* for ligands and *k* for the transition metal ion, which have been used to calculate the ratio of Racah *B*/*B*_0_ parameter for octahedral coordination compounds. According to this relation, if the lone pair of the ligand penetrates the d orbitals, they will tend to screen the d electrons from the nucleus, decrease the effective charge experienced by the d electrons, and expand the d shell. Consequently, the Racah *B* parameter is expected to decrease if the metal ligand bond becomes partially covalent.^47^ Nevertheless, the final results regarding the Ln/An-ligand bonds were still qualitative in nature, despite being based on the spectroscopic data.

The X-ray electron density distribution method (hereinafter termed XREDD) is in fact one of the few robust experimental tools that may provide direct information concerning the nature of chemical bonds. Thus, the parameters such as the electron density (ρ_c_) and Laplacian (∇^2^ρ_c_) in the bond critical point and energy densities, as well as bond degrees (BD), which are derived directly from the above-mentioned parameters, are particularly useful to describe the multifaceted problem of the bond covalency. According to Espinosa et al., a bond may be classified as a shared shell (SS) one if both ∇^2^ρ_c_ and the bond degree (BD) values are less than zero.^48^ Such a bond may be interpreted as being covalent. A bond is classified as transition closed shell (TCS) when ∇^2^ρ_c_ > 0 and BD < 0; such a bond may be interpreted as polarized covalent. The closed shell (CS) or ionic interaction is the case when both ∇^2^ρ_c_ and BD are positive. Previous results concerning experimental electron density distribution analysis in [Gd(H_2_O)_9_](CF_3_SO_3_)_3_ crystal, supported additionally by the theoretical DFT calculations, enabled us to conclude that the Gd(III)–OH_2_ interactions may be classified as polarized covalent bond (TCS). The electron density of sp^*x*^ molecular orbitals of the oxygen atoms of water is shifted mainly to the 6s and 5d orbitals of Gd(III). Accordingly, the configuration of the Gd(III) in that compound was [Xe]6s^0.164^f^7.105^d^0.69^.^49^ An interesting result was obtained by Ungur and Chibotaru, namely, they have shown that the exact space distribution of the electronic charge in the ligands may significantly improve calculated values of the CF levels in comparison to those obtained within purely electrostatic model, as was shown for the Er(III) complexes with 2,2′,2″-tris(salicylideneimido)trimethylamine.^50^ In light of the aforementioned results, it becomes appropriate to inquire as to whether there exists a correlation between the parameters derived from XREDD and high-resolution electronic spectroscopy of f–f transitions. Moreover, is there any physical property which may reflect the changes of bond covalency in Ln(III) compounds? To the best of our knowledge, such correlations have not been studied so far.

Accordingly, the objective of this Letter is to investigate the spectroscopic properties of a series of Gd(III) coordination compounds with common polyaminopolycarboxylates (EDTA, NTA, EGTA, DTPA, DOTA, CDTA, ODA)^51^ and/or inorganic ligands (CO_3_^2–^, F^–^, H_2_O), in the context of electron density distribution derived for [C(NH_2_)_3_]_3_[Gd(EDTA)F_2_]·H_2_O and also with previously published results for [GdCl(*o*-phenanthroline)_2_(H_2_O)_3_]Cl_2_(H_2_O)^52^ and [Gd(H_2_O)_9_](CF_3_SO_3_)_3_ crystals.^49^ The reason for selection of this particular group of the Gd(III) compounds is the fact that they are or may be used as MRI contrast agents. Moreover, the Gd(III) cation which is not spectroscopically silent in UV region owing to partially filled 4f^7^ configuration is simultaneously spherically symmetric; thus, it is very suitable for XREDD analyses. The XREDD results will be augmented by the DFT calculations to finally find the correlation between the f–f transition spectra, Gd–L bond lengths and energy, and donor properties of the coordinated ligands.

*Crystal Structures*. Crystals with the formulas [C(NH_2_)_3_]_3_[Gd(EDTA)F_2_]·H_2_O, [C(NH_2_)_3_]_3_[Gd(DTPA)F]·H_2_O, [C(NH_2_)_3_]_3_[Gd(CDTA)(CO_3_)]·H_2_O, and Na_2_[Gd(DTPA)(H_2_O)]·7.875H_2_O were obtained. Drawings of the respective complex anions are shown in Figure 1.

**Figure 1:**
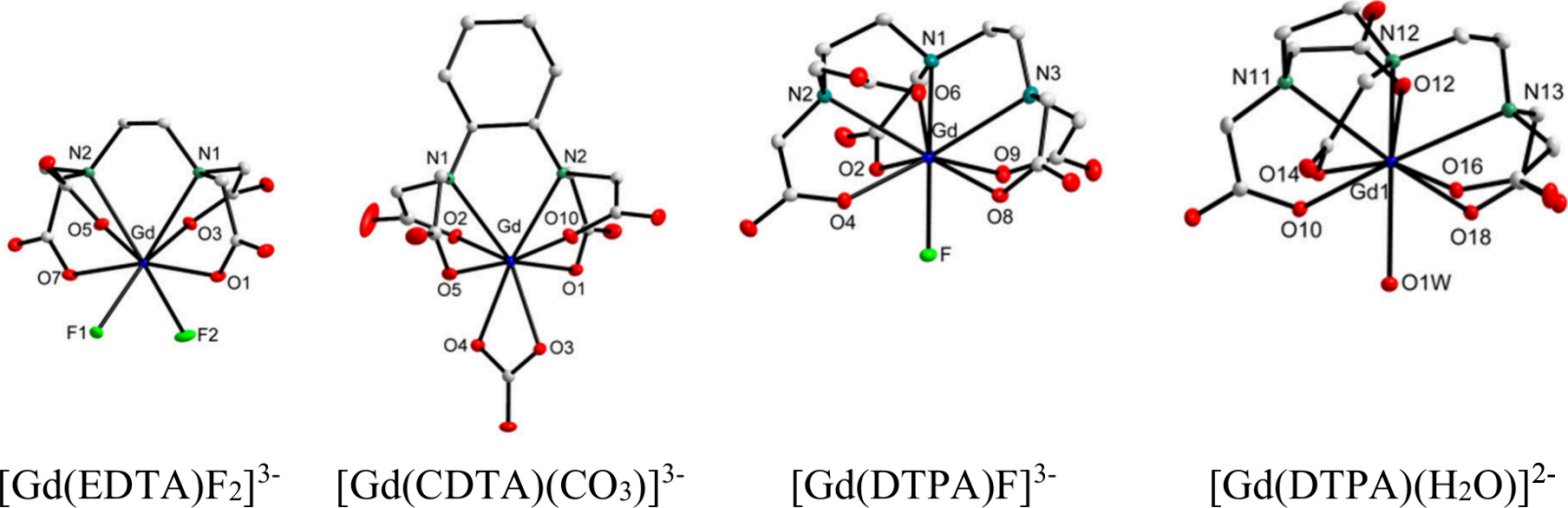
A DIAMOND view of the complexes [Gd(EDTA)F_2_]^3–^, [Gd(CDTA)(CO_3_)]^3–^, [Gd(DTPA)F]^3–^, and [Gd(DTPA)(H_2_O)]^2–^ anions. For the sake of clarity, the H atoms have been omitted, and the labels are given for non-C atoms only.

The crystal of [C(NH_2_)_3_]_3_[Gd(EDTA)F_2_]·H_2_O is isostructural with that previously published.^53^ The EDTA and CDTA ligands are coordinated to Gd(III) by four oxygen atoms from carboxyl groups and two nitrogen atoms. The coordination sphere the Gd(III) is completed by two fluoride anions in the [Gd(EDTA)F_2_]^3–^ and two oxygen atoms from the bidentate carbonate anion in the [Gd(CDTA)(CO_3_)]^3–^ anion. Thus, the Gd(III) cation has coordination number 8 in both compounds. The DTPA anions in the [Gd(DTPA)F]^3–^ and [Gd(DTPA)(H_2_O)]^2–^ complex anions coordinate to the Gd(III) in a similar way, namely by five monodentate carboxylate groups and three nitrogen atoms, and additionally the Gd(III) coordination sphere is completed by a fluoride anion or a water molecule, respectively. In the Na_2_[Gd(DTPA)(H_2_O)]·7.875H_2_O crystal there are 4 symmetry-independent complex anions. The lengths of bonds formed by Gd(III) are given in Table 1.

**Table 1:** Gd–L Bond Lengths (in Å)

[Gd(EDTA)F_2_]^3–^		[Gd(CDTA)(CO_3_)]^3–^		[Gd(DTPA)F]^3–^	
Gd–O1	2.3670(5)	Gd–O10	2.3088(14)	Gd–O6	2.370(2)
Gd–O3	2.3722(5)	Gd–O1	2.3622(14)	Gd–O9	2.389(2)
Gd–O5	2.3610(5)	Gd–O2	2.3631(13)	Gd–O2	2.399(2)
Gd–O7	2.3828(6)	Gd–O5	2.3781(15)	Gd–O8	2.425(2)
Gd–F1	2.2553(8)	Gd–O4	2.3821(14)	Gd–O4	2.483(2)
Gd–F2	2.2252(9)	Gd–O3	2.3859(13)	Gd–F1	2.209(2)
Gd–N1	2.6654(4)	Gd–N2	2.5716(16)	Gd–N1	2.638(2)
Gd–N2	2.6386(4)	Gd–N1	2.5857(16)	Gd–N2	2.723(2)
				Gd–N3	2.676(2)
[Gd(DTPA)(H_2_O)]^2–^					
Gd1–O12	2.3259(17)	Gd2–O26	2.4138(16)	Gd3–N32	2.5938(18)
Gd1–O14	2.3572(16)	Gd2–O22	2.4431(16)	Gd3–N33	2.6491(18)
Gd1–O18	2.4151(16)	Gd2–O2W	2.4758(16)	Gd3–N31	2.6984(18)
Gd1–O10	2.4172(17)	Gd2–N22	2.6071(19)	Gd4–O42	2.3601(16)
Gd1–O16	2.4339(17)	Gd2–N21	2.6143(19)	Gd4–O44	2.3733(16)
Gd1–O1W	2.5159(16)	Gd2–N23	2.7292(19)	Gd4–O40	2.4048(17)
Gd1–N12	2.6078(19)	Gd3–O32	2.3720(16)	Gd4–O46	2.4082(16)
Gd1–N13	2.6200(19)	Gd3–O34	2.3856(16)	Gd4–O48	2.4109(16)
Gd1–N11	2.7246(19)	Gd3–O36	2.4065(16)	Gd4–O4W	2.4815(16)
Gd2–O28	2.3498(17)	Gd3–O38	2.4144(16)	Gd4–N42	2.5935(19)
Gd2–O20	2.4015(16)	Gd3–O30	2.4275(16)	Gd4–N43	2.6256(19)
Gd2–O24	2.4044(16)	Gd3–O3W	2.4847(16)	Gd4–N41	2.6785(19)

Interestingly, the Gd–F bond in the 9-coordinate [Gd(DTPA)F]^3–^ anion is shorter by an average 0.031 Å than in the 8-coordinate [Gd(EDTA)F_2_]^3–^. This fact prompted us to investigate the nature of the Gd–F interaction in more detail. Out of the prepared crystals, [C(NH_2_)_3_]_3_[Gd(EDTA)F_2_]·H_2_O turned out to be the most stable. Moreover, the Gd(III) coordination sphere is characterized by the greatest chemical variety of donor atom ligands; this compound seemed therefore to be the most suitable for studying the X-ray electron density distribution.

*Electron Density Analysis of [C(NH_2_)_3_]_3_[Gd(EDTA)F_2_]·H_2_O*. The refinement has yielded rather satisfactory general parameters; nevertheless, there have remained regions of rather large unaccounted density in the residual map. The most problematic ones are located near Gd (see Figure S1). The residual density is more pronounced than that reported in the case of nonaaquagadolinium tris(trifluoromethanesulfonate).^49^ It has been observed previously^54^ that analysis of the experimental charge density of systems with lanthanides is a challenging task; the features observed in the residual density maps are largely dependent upon the lanthanide scattering factors chosen. Apart from that the influence of small imprecision of crystal shape definition, used for calculation of absorption, cannot be excluded, either.

*The Bader Charges*. The Bader charges for Gd coordination environment, both experimental and theoretical, are collected in Table 2.

**Table 2:** Bader Charges for Gd and Its Coordination Surroundings^a^

atom	*Q*_exp_	*Q*_1_	*Q*_2_
Gd	2.49	2.18	2.18
F1	–0.72	–0.79	–0.86
F2	–0.58	–0.79	–0.86
O1	–1.15	–1.21	–1.21
O3	–1.25	–1.21	–1.21
O5	–1.16	–1.19	–1.18
O7	–1.25	–1.22	–1.22
N1	–0.84	–0.90	–0.89
N2	–0.85	–0.91	–0.90

a *Q*_exp_, charges derived from the experimental charge distribution; *Q*_1_, theoretical charges obtained for the {[Gd(EDTA)F_2_][C(NH_2_)_3_]_4_}^+^ cluster; and *Q*_2_, theoretical charges retrieved from the calculations of the [Gd(EDTA)F_2_]^3–^ anion.

The overall agreement between the experimental and theoretical values is satisfactory, except for the case of F2, where the theoretical charges are more than 0.2*e* smaller than the experimental one; the reason for this is not completely clear. The Gd charges are in better agreement than those found in Gd triflate; moreover, they tend to be slightly smaller than those reported for the quoted compound. During various attempted refinements, we have found, what should be stressed, that the experimental charge of Gd is strongly dependent upon the parametrization selected for the description of the atom. As far as the F anions are concerned, it may be somewhat surprising to find that their charges are larger (i.e., less negative) than those of the carboxylate O atoms. The charges are intermediate between those found in KMnF_3_ at 0.3 GPa^55^ (−0.96) and in CaF_2_^56^ (−0.70). The present charges of the carboxylate O atoms are typical for other ionized carboxylate groups, e.g., −1.16^57^ or −1.10.^58^

*Topology*. Experimental and theoretical topological parameters for Gd–F,O,N bonds and F···H hydrogen bonds are given in Table 3. The experimental parameters, with the exception of the ellipticities of the F···H hydrogen bonds, are in satisfactory agreement with the theoretical parameters, although the experimental values seem to be generally slightly underestimated. Data regarding the topological parameters of bonds formed by lanthanides are scarce. The values of ρ_*c*_ for Gd–O bonds are similar to those for the shorter Gd–O bonds in the complex [Gd(H_2_O)_9_]^3+^ cation,^49^ whereas the laplacian values are slightly higher, thus resembling values reported for such bonds in [GdCl(*o*-phenanthroline)_2_(H_2_O)_3_]Cl_2_(H_2_O).^52^

**Table 3:** Topological Parameters for Bonds Involving Gd and Hydrogen F···H Bonds^a^

	experimental			theoretical^b^		
	ρ_c_ (eÅ^–3^)	∇^2^ρ_c_ (eÅ^–5^)	ε	ρ_c_ (eÅ^–3^)	∇^2^ρ_c_ (eÅ^–5^)	ε
Gd–F1	0.365(1)	6.106(7)	0.01	0.428	7.427	0.01
				*0.441*	*7.257*	*0.02*
Gd–F2	0.388(1)	6.663(8)	0.00	0.464	8.037	0.01
				*0.474*	*7.869*	*0.01*
Gd–O1	0.311(2)	4.482(6)	0.01	0.375	5.353	0.05
				*0.369*	*5.417*	*0.04*
Gd–O3	0.309(2)	4.452(7)	0.01	0.366	5.343	0.04
				*0.367*	*5.352*	*0.03*
Gd–O5	0.317(2)	4.612(6)	0.01	0.381	5.404	0.02
				*0.375*	*5.500*	*0.02*
Gd–O7	0.303(2)	4.377(6)	0.01	0.363	5.156	0.04
				*0.356*	*5.219*	*0.03*
Gd–N1	0.225(3)	2.383(2)	0.03	0.235	2.587	0.03
				*0.231*	*2.653*	*0.03*
Gd–N2	0.234(3)	2.513(2)	0.05	0.250	2.735	0.02
				*0.245*	*2.814*	*0.02*
F1···H12A^c^	0.29(10)	2.27(15)	0.19	0.280	3.147	0.03
F1···H33A^d^	0.19(7)	2.82(10)	0.76	0.237	2.751	0.01
F1···H2W^e^	0.22(8)	2.65(16)	0.36	0.261	3.105	0.03
F2···H11A^c^	0.19(5)	2.80(5)	0.19	0.229	2.650	0.00
F2···H12B^f^	0.21(7)	3.96(11)	0.82	0.293	3.188	0.02

a ρ_c_ is the electron density at the bond critical point, ∇^2^ρ_c_ is the laplacian, and ε is ellipticity.

b Parameters retrieved from the {[Gd(EDTA)F_2_][C(NH_2_)_3_]_4_}^+^ cluster are given in the first line; those obtained from [Gd(edta)F_2_]^3–^ anion are in the second line in italics.

c Symmetry codes: −1/2–*x*, −1/2+*y*, 1/2–*z*.

d –1/2+*x*, 3/2–*y*, −1/2+*z*.

e 1/2–*x*, 1/2+*y*, 1/2–*z*.

f *x*, −1+*y*, *z*.

On the other hand, both ρ_c_ and ∇^2^ρ_c_ parameters for Gd-N bonds are slightly smaller than in the case of the Gd-phenanthroline complex (ρ_c_ = 0.27–0.29 eÅ^–3^, ∇^2^ρ_c_ = 3.48–3.66 eÅ^–5^). It was difficult to find data concerning the Gd–F bonds; it must therefore suffice to observe that both the densities and laplacians at the critical points for these bonds are somewhat larger than those of Gd–O bonds.

The deformation density and laplacian maps for the selected bonds are presented in Figure 2. It may be noticed that the fluoride anion is completely unpolarized and the oxygen atoms are only slightly polarized, which may be shown more convincingly in the deformation density maps; the lone electron pair of N1 is distinctly visible in both maps.

**Figure 2:**
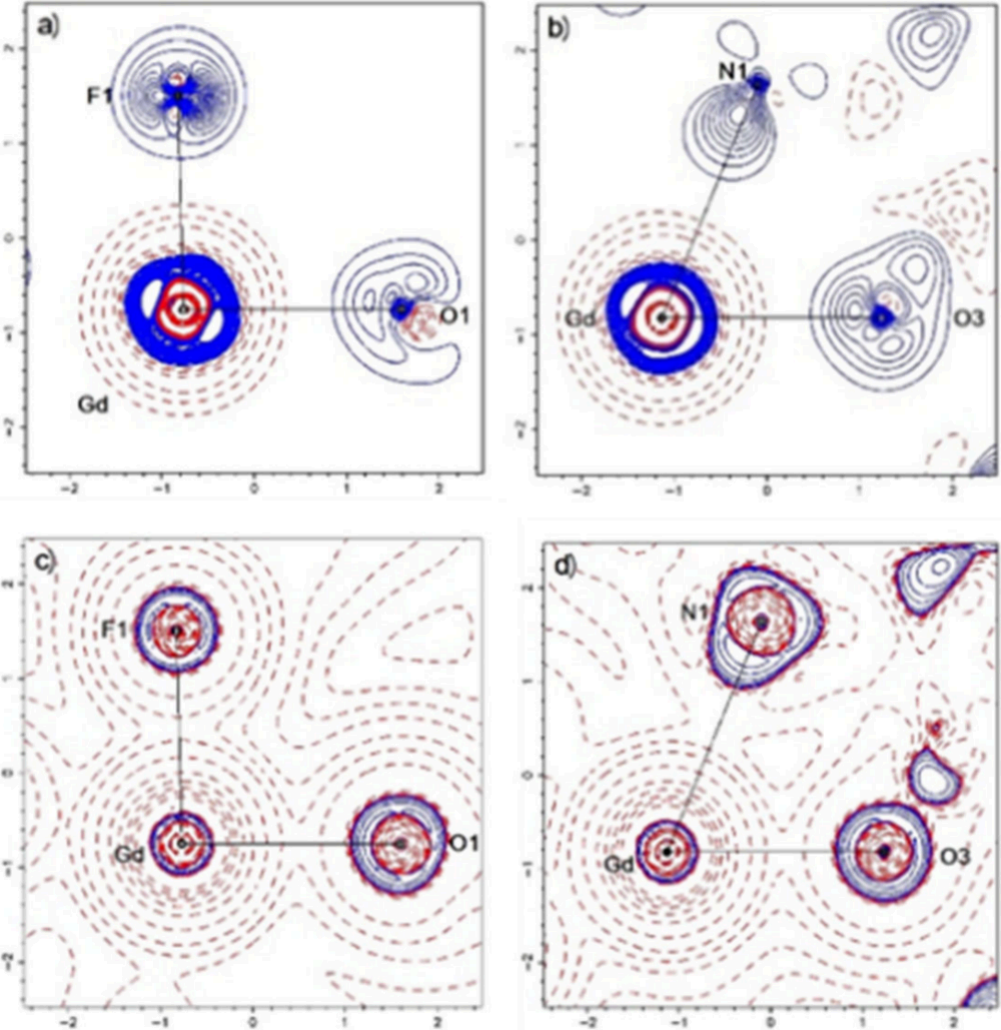
Deformation density maps: (a) section through F1, Gd, and O1; (b) section through N1, Gd, and O3; the contours (positive solid blue, negative dashed red) are drawn every 0.1 eÅ^–3^. Laplacian maps: (c) section through F1, Gd, and O1; (d) section through N1, Gd and O3; the positive contours are dashed red and the negative are solid blue.

Topological criteria may be used to classify atomic interactions. Espinosa et al.^48^ divided them into three classes: (a) shared shell interactions, SS, when ∇^2^ρ_c_ < 0; (b) transit closed shell ones, TCS, if ∇^2^ρ_c_ > 0 and BD < 0; (c) closed shell ones, CS, if ∇^2^ρ_c_ > 0 and BD > 0. For the discussion of bonds formed by Gd we shall interpret the SS interactions as covalent ones, TCS as polarized covalent, and CS as ionic. To get insight into the character of these bond, relevant parameters (energy densities and bond degrees) are collected in Table 4.

**Table 4:** Experimental and Theoretical Energy Densities at Bond Critical Points and Bond Degrees for Bonds Formed by Gd^a^

	experimental					theoretical^b^				
bond	*G*(r_c_)	*V*(r_c_)	*E*(r_c_)	BD	type	*G*(r_c_)	*V*(r_c_)	*E*(r_c_)	BD	type
Gd–F1	0.064	–0.066	–0.001	–0.017	TCS	0.080	–0.084	–0.003	–0.052	TCS
						*0.081*	*–0.086*	*–0.005*	*–0.082*	*TCS*
Gd–F2	0.071	–0.072	–0.002	–0.022	TCS	0.089	–0.094	–0.005	–0.077	TCS
						*0.089*	*–0.096*	*–0.007*	*–0.102*	*TCS*
Gd–O1	0.048	–0.050	–0.002	–0.031	TCS	0.060	–0.065	–0.005	–0.085	TCS
						0.060	0.064	–0.004	–0.071	*TCS*
Gd–O3	0.048	–0.049	–0.001	–0.030	TCS	0.059	–0.063	–0.004	–0.072	TCS
						*0.059*	*–0.063*	*–0.004*	*–0.071*	*TCS*
Gd–O5	0.049	–0.051	–0.002	–0.033	TCS	0.061	–0.066	–0.005	–0.092	TCS
						*0.061*	*–0.066*	*–0.004*	*–0.077*	*TCS*
Gd–O7	0.047	–0.048	–0.001	–0.025	TCS	0.058	–0.062	–0.004	–0.077	TCS
						*0.057*	*–0.061*	*–0.003*	*–0.062*	*TCS*
Gd–N1	0.026	–0.028	–0.002	–0.064	TCS	0.029	–0.030	–0.002	–0.050	TCS
						*0.029*	*–0.030*	*–0.001*	*–0.034*	*TCS*
Gd–N2	0.028	–0.030	–0.002	–0.068	TCS	0.031	–0.033	–0.002	–0.063	TCS
						*0.031*	*–0.033*	*–0.002*	*–0.046*	*TCS*

a All quantities are in atomic units.

b Parameters retrieved from the {[Gd(EDTA)F_2_][C(NH_2_)_3_]_4_}^+^ cluster are given in the first line; those obtained from [Gd(EDTA)F_2_]^3–^ anion are in the second line in italics.

Inspection of this table shows that all the bonds are of the TCS type, which may be interpreted as the bonds, from the topological point of view, are strongly polarized covalent ones. Moreover, the parameters for the Gd–F and Gd–O interactions are essentially similar.

*Electronic Structure of [Gd(EDTA)F_2_]^3–^*. The results presented here will be based on the NBO approach, which basically allows analyzing the bonds in two ways. The first method consists of inspecting the NBO orbitals, which are strictly 1- or 2-centered but with noninteger occupations; the interactions may be monitored here by examining the population depletion of the valence orbitals and associated populating of empty virtual ones. The other method focuses on analysis of the natural localized molecular orbitals (NLMO) which have strict integer occupations, but their strict localization is perturbed by the admixture of wave functions from neighboring atoms; these admixtures are inspected.

To begin, some general information will be useful. The natural charge of Gd is 1.86, and the natural configuration of the cation is [Xe]6s^0.13^4f^7.10^5d^0.83^. In the cited Gd triflate the charge was 1.96 and the configuration was [Xe]6s^0.16^4f^7.10^5d^0.69^; thus, in the present case the transfer of charge onto the metal is greater and it is directed predominantly to the 5d orbitals of Gd. The Wiberg indices of bonds formed by gadolinium are the following: Gd–F1, 0.37; Gd–F2, 0.39; Gd–O1, 0.26; Gd–O3, 0.25; Gd–O5, 0.25; Gd–O7, 0.26; Gd–N1, 0.15; and Gd–N2, 0.16. Thus the values for the Gd–F and Gd–O bonds are greater than for the [Gd(H_2_O)_9_](CF_3_SO_3_)_3_ (Gd–O, 0.22 and 0.18). The low value for the Gd–N bonds may be a result of larger Gd–N bond lengths in comparison with Gd–O and Gd–F. The NBO spin-orbitals of interest are collected in Table 5.

**Table 5:** Lone Pairs of Ligating Atoms and Virtual 5d and 6s Spin-Orbitals of Gd^a^

	hybridization	population	Σ*E*(2) (kcal/mol)
atom	spin α/β	spin α/β	spin α/β
F1	sp^1.40^/sp^1.25^	0.992/0.992	8.75/10.82
	p/p	0.982/0.976	5.38/4.44
	p/p	0.977/0.972	5.16/5.21
	sp^0.72^/sp^0.80^	0.955/0.949	40.18/37.51
F2	sp^1.44^/sp^1.29^	0.991/0.991	8.88/11.21
	p/p	0.980/0.974	5.79/4.82
	p/p	0.977/0.971	5.83/5.46
	sp^0.70^/sp^0.78^	0.954/0.948	41.34/38.96
O1	sp^0.92^/sp^0.95^	0.963/0.961	21.01/20.28
	sp^6.38^/sp^6.02^	0.930/0.924	28.64/25.88
	p/p	0.817/0.815	3.06/3.30
O3	sp^0.89^/sp^0.90^	0.965/0.962	13.17/13.69
	sp^7.10^/sp^6.73^	0.931/0.924	18.13/17.37
	p/p	0.817/0.815	4.25/3.57
O5	sp^0.92^/sp^0.94^	0.964/0.962	12.69/13.43
	sp^6.23^/sp^5.98^	0.931/0.924	19.80/19.32
	p/p	0.819/0.816	3.19/2.78
O7	sp^0.91^/sp^0.94^	0.963/0.961	20.40/19.75
	sp^6.80^/sp^6.34^	0.929/0.923	28.52/25.70
	p/p	0.815/0.813	2.77/2.91
N1	sp^3.73^/sp^3.77^	0.910/0.905	13.98/12.32
N2	sp^3.62^/sp^3.66^	0.910/0.905	14.87/13.03
Gd	d/d	0.107/0.101	
	d/d	0.097/0.091	
	d/d	0.091/0.088	
	d/d	0.085/0.081	
	s/s	0.065/0.062	
	d/d	0.055/0.052	

a The sum second-order stabilization energy (Σ*E*) is the sum of the interactions with the 5d and 6s spin-orbitals of Gd.

This table includes also the second-order donor–acceptor stabilization energies between the lone pairs of the ligating atoms and the 5d and 6s virtual spin-orbitals, which are defined as
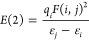
1where *q*_*i*_ is the occupation of donor (spin)orbital; *F*(*i*, *j*) is the NBO Fock matrix element; and ε_*j*_ and ε_*i*_ are the energies of acceptor and donor (spin)orbitals, respectively.

The data presented in Table 5 show that in the Gd–ligand interactions sp^*x*^ hybrids play a dominant role, in rough accordance with what was observed for the nonaaquagadolinium cation. The share of the s function, however, is greater in the case of fluoride anions, where it tends to prevail, while for the lone pairs of the O atoms the p character predominates. The depopulation degrees are slightly larger, and the *E*(2) values of the sp^*x*^ spin-orbitals of the coordinated O atoms are similar to those of the cited cation (where the populations were around 0.96 and sum *E*(2) ― 25.4–27.6 kcal/mol). Depopulation of the *p*-type orbitals is the result of the delocalization of electrons within the carboxylate group. As far as the fluoride anions are concerned, their basic hybridization may be described as *sp*, and one of the hybrids (or to be more precise, one pair of α and β spin-orbitals) is involved in relatively strong interaction with the empty 5d and 6s orbitals of Gd; both the value of population of such spin-orbitals (which is lower than the relevant quantity for the O atoms) and the high values of Σ*E*(2) show that the covalent Gd–F interactions are stronger than the Gd–O ones, not to mention the rather weak Gd–N bonds. Exemplary overlapping of sp^*x*^ spin-orbitals of F and O with 5d ones of Gd are presented in Figure 3.

**Figure 3:**
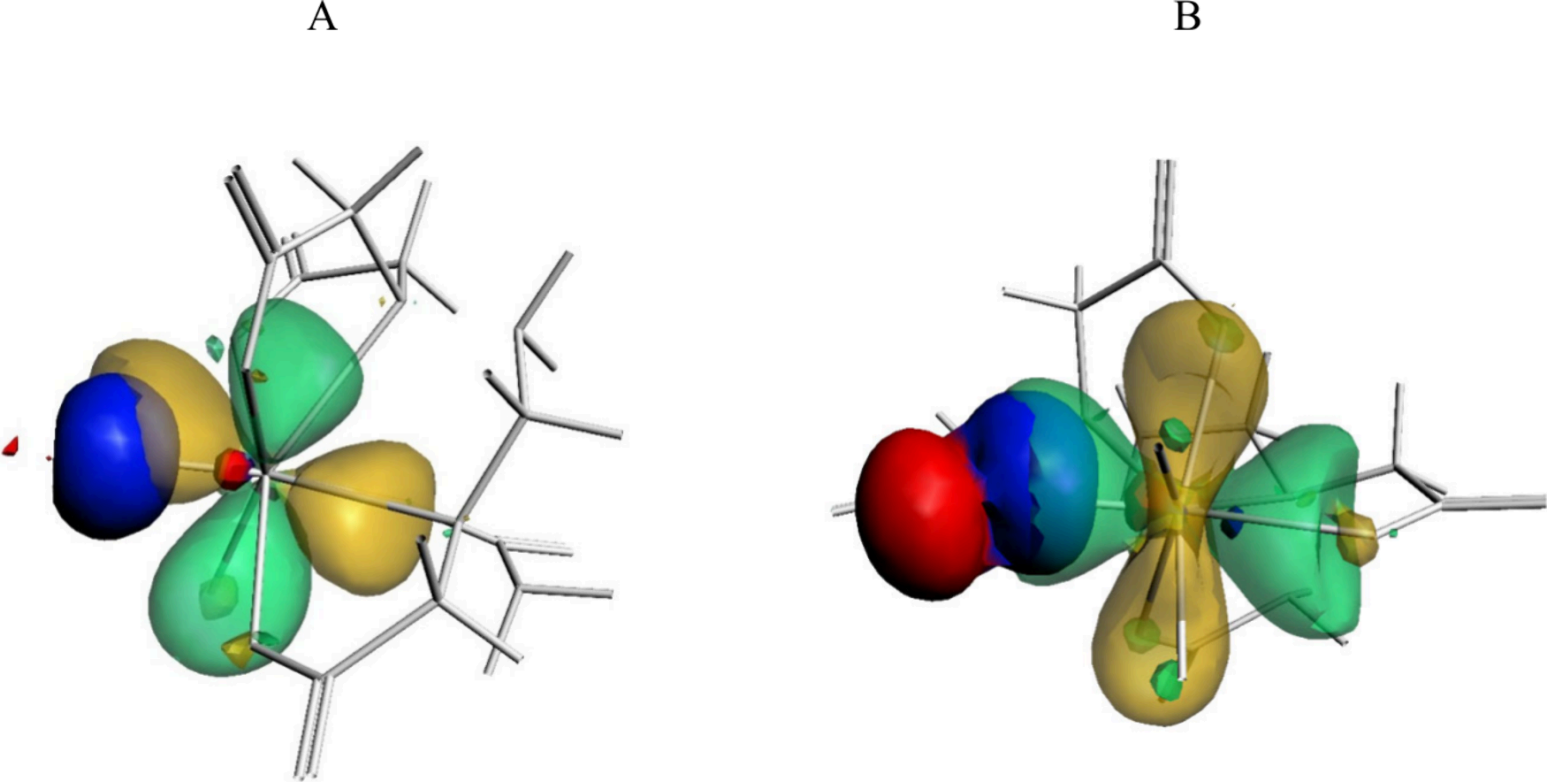
(A) Overlapping of the α sp^0.69^ spin-orbital of F2 (red and blue) with a 5d spin-orbital of Gd (green and yellow, transparent); (B) overlapping of the α sp^6.38^ spin-orbital of O1 (red and blue) with a 5d spin-orbital of Gd (green and yellow, transparent).

A complementary view of the interactions of Gd may be gained by examining the composition of the NLM spin-orbitals of the ligand atoms. Relevant data are listed in Table 6. It may be inferred from the presented results that the 5d spin-orbitals of Gd are the main donor of the electrons to the NLM spin-orbitals of the ligands; a secondary role is played by the 6s spin-orbitals, and in the case of β spin electrons, the 4f ones. The admixtures are not large, but they are, nevertheless, larger than those calculated for the nonaaquagadolinium cation.^49^ Out of the coordinated atoms, the NLM spin-orbitals of the fluoride anions exercise the strongest influence of the Gd functions.

**Table 6:** NLM Lone-Pair Spin Orbitals of the Ligating Atoms^a^

atom	basic spin-orbital	admixture composition	admixture contribution (%)
spin α			
F1	p	d	2.17
	sp^0.71^	sd^3.47^	4.46
F2	p	d	2.25
	sp^0.69^	sd^3.84^	4.62
O1	sp^6.06^	sd^5.96^	3.68
O3	sp^6.75^	sd^5.58^	3.26
O5	sp^5.83^	sd^5.23^	3.43
O7	sp^6.36^	sd^5.63^	3.46
N1	sp^3.93^	sd^11.59^	3.62
N2	sp^3.79^	sd^11.75^	3.66
spin β			
F1	p	df^0.54^	2.23
	p	df^0.43^	2.69
	sp^0.80^	sd^3.35^f^1.11^	5.03
F2	p	df^0.50^	2.53
	p	df^0.45^	2.77
	sp^0.69^	sd^3.75^f^1.21^	5.20
O1	sp^0.98^	sd^3.52^f^0.76^	2.02
	sp^5.65^	sd^5.71^f^2.21^	4.45
O3	sp^6.36^	sd^5.56^f^2.35^	4.04
O5	sp^5.59^	sd^5.26^f^2.31^	4.24
O7	sp^5.95^	sd^5.33^f^2.27^	4.27
N1	sp^3.95^	sd^12.00^f^3.17^	4.21
N2	sp^3.81^	sd^10.85^f^3.10^	4.24

a Only those with Gd function admixture greater than 2% are listed.

*UV Spectroscopy of 4f^7^–4f^7^ Transitions of the Gd(III) Compounds*. The aforementioned XREDD parameters together with those published previously for [GdCl(*o*-phenanthroline)_2_(H_2_O)_3_]Cl_2_(H_2_O)^52^ and [Gd(H_2_O)_9_](CF_3_SO_3_)_3_^49^ were used to find a correlation with the spectroscopic figures, such as energy of the selected ^8^S_7/2_ → ^2*S*+1^L_J_ bands and Judd-Ofelt (Ω_λ_) intensity parameters, luminescence lifetimes, etc. The crystalline systems containing Gd(III) molecular complexes given in Table 7 were analyzed.

**Table 7:** Gd(III) Coordination Compounds under Study Together with the Gd(III) Coordination Environment^a^

	Compound	Coordination environment	ref.
1	[Gd(H_2_O)_8_]Cl_3_·C_10_H_20_O_5_	O_8_	(38)
2	[Lu:Gd(H_2_O)_8_]Cl_3_·C_12_H_24_O_6_	O_8_	(38)
3	[Gd(H_2_O)_9_](CF_3_SO_3_)_3_	O_9_	(38)
4	[C(NH_2_)_3_]_5_[Gd(CO_3_)_4_(H_2_O)]·2H_2_O	O_8_^′^O_1_	This study
5	[C(NH_2_)_3_]_5_[Y:Gd(CO_3_)_4_]·2H_2_O	O_8_^′^	This study
6	[C(NH_2_)_3_][Gd(EDTA)(H_2_O)_3_]	O_4_^″^O_3_N_2_	(38)
7	Na[Gd(EDTA)(H_2_O)_3_]·H_2_O	O_4_^″^O_3_N_2_	This study
8	[C(NH_2_)_3_]_2_[Lu:Gd(EDTA)(H_2_O)_2_]ClO_4_·4H_2_O	O_4_^″^O_2_N_2_	(38)
9	[C(NH_2_)_3_]_3_[Gd(EDTA)F_2_]·H_2_O	F_2_ O_4_^″^N_2_	This study
10	[C(NH_2_)_3_]_3_[Gd(CDTA)(CO_3_)]·H_2_O	O_4_^″^O_2_^′^N_2_	This study
11	[C(NH_2_)_2_(N_2_H_4_)][Gd(HDTPA)(H_2_O)]·2H_2_O	O_5_^″^O_1_N_3_	(38)
12	Na[Gd(DTPA)(H_2_O)] ·7.875H_2_O	O_5_^″^O_1_N_3_	This study
13	[C(NH_2_)_3_]_3_[Gd(DTPA)F]·H_2_O	F_1_O_5_^″^N_3_	This study
14	[C(NH_2_)_3_][Gd(EGTA)(H_2_O)]·2H_2_O	O_2_^‴^O_4_^″^O_1_N_2_	(38)
15	Na[Gd(DOTA)(H_2_O)]·4H_2_O	O_4_^″^O_1_N_4_	(38)
16	K[Lu:Gd(DOTA)]·KCl·4.6H_2_O	O_4_^″^N_4_	(38)
17	K_3_[Gd(NTA)_2_(H_2_O)]	O_6_^″^O_1_N_2_	This study
18	Na_3_[Gd(ODA)_3_]·2NaClO_4_·6H_2_O	O_3_^‴^O_6_^″^	(59)

a O -O_water_; O′ -O_carbonate_; O″ -O_caboxylate_; O‴ -O_ether_.

The UV electronic spectra of Gd(III) ion consist of narrow low-intensity bands attributed to the intraconfigurational 4f^7^–4f^7^ transitions. In the spectral range between 315 and 215 nm, 8 bands, attributed to the transitions ^8^S_7/2_ → ^6^P_7/2_ (∼310 nm); ^8^S_7/2_ → ^6^P_5/2_ (∼305 nm); ^8^S_7/2_ → ^6^P_3/2_ (∼300 nm); ^8^S_7/2_ → ^6^I_7/2_ (∼278 nm); ^8^S_7/2_ → ^6^I_9/2_,^6^I_17/2_ (∼276 nm); ^8^S_7/2_ → ^6^I_11/2_,^6^I_13/2_,^6^I_15/2_ (∼271 nm); ^8^S_7/2_ → ^6^D_9/2_ (∼251 nm); and ^8^S_7/2_ → ^6^*D*_1/2_,^6^D_7/2_,^6^D_3/2_,^6^D_5/2_ (∼245 nm), are observed. Below 240 nm, the 4f^7^–4f^7^ bands specific for the Gd(III) ion in molecular compounds may usually be superimposed on ligand/counterion bands. For these reasons, we have decided to analyze systems in which the ligands/counterions are practically spectroscopically silent in the UV range.

Out of the aforementioned bands, only the ^8^S_7/2_ → ^6^P_7/2_, ^8^S_7/2_ → ^6^P_5/2_, ^8^S_7/2_ → ^6^P_3/2_, ^8^S_7/2_ → ^6^I_7/2_, and ^8^S_7/2_ → ^6^D_9/2_ ones are well separated from each other, and thus, their gravity center (hereinafter referred to as ν̅_GC_) may be determined undoubtedly. The ν̅_GC_ as well as crystal field splitting (CFS) values are presented in Table S2. As was shown by Antic-Fidancev,^60^ the energies of ^8^S_7/2_ → ^6^P_7/2_ and ^8^S_7/2_ → ^6^P_5/2_ bands are linearly dependent. We have also observed such a strong correlation ν̅_GC_ of the ^8^S_7/2_ → ^6^P_7/2_ band energy and the other ^8^S_7/2_ → ^6^P_5/2_, ^8^S_7/2_ → ^6^P_3/2_, ^8^S_7/2_ → ^6^I_7/2_, and ^8^S_7/2_ → ^6^D_9/2_ ones. Since the present compounds are characterized by low symmetry (except of [Gd(OH_2_)_9_]^3+^), it can be assumed that the shift of the centers of gravity is in fact dependent only on the type of coordinated atoms and their distances from the central Gd(III) cation. Such an assumption seems to be justified because largest crystal field splitting (hereinafter referred to as CFS) values of the ^6^P_7/2_, ^6^P_5/2_, ^6^P_3/2_, ^6^I_7/2_, and ^6^D_9/2_ multiplets (collected in Table S2) in the spectra of compounds under study are 128, 112, 44, 129, and 184 cm^–1^, respectively, while the shifts of the gravicenters of these states in comparison with gaseous Gd(IV)^61^ are almost 2.5 times higher (290–330 cm^–1^). Moreover, it is likely that the excited states are more sensitive to the changes of the coordinated ligands than the ground ^8^S_7/2_ state, because the 4f^7^ electrons are distributed spherically in the ground state and the spherical symmetry is more or less lost in the excited states.

To find the correlation between the number of donor atom, the Gd–L bond lengths, and the ν̅_GC_ values, different approaches were considered, and finally the best fit was obtained for the following expression:
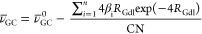
2where *v̅*_GC_^0^ is energy of the respective excited manifolds of the Gd(III) free ion, CN is the coordination number, *n* is the number of coordinated atoms, β_i_ is the fitted partial nephelauxetic parameter for a given donor atom, and *R*_GdL_ is the Gd–L bond length in Å.

The linear regression enabled us to determine the energy values of the selected multiplets in the free Gd(III) ion as well as the nephelauxetic parameters. The calculated *v̅*_GC_^0^ vales are in excellent agreement with their counterparts for gaseous Gd(III) free ion taken from NIST (Table 8).^62^

**Table 8:** Experimental (NIST) and Predicted Values of the Selected ^8^S_7/2_ → ^2*S*+1^L_J_ Transitions

	NIST^62^	This study	|Δ|
^8^S_7/2_→^6^P_7/2_	32120	32498	378
^8^S_7/2_→^6^P_5/2_	32720	33089	369
^8^S_7/2_→^6^P_3/2_	33290	33662	372
^8^S_7/2_→^6^I_7/2_		36043	
^8^S_7/2_→^6^D_9/2_		39770	

The sign of the calculated nephelauxetic parameters is positive as formation of the Gd(III)–L bond decreases the energies of the ^2*S*+1^L_J_ states relative to those in gaseous Gd(III) ion (Table 9). The mean standard error of the energy estimation for the considered multiplets was found to be δν̅ = 13 cm^–1^.

**Table 9:** Fitted Partial Nephelauxetic β_i_ for the Donor Atoms (i), Diffusive  (*Q*, Bader charge; χ, electronegativity difference of Gd and donor ligand), and *a,b* (eq 3) parameters

Donor ligand	β_i_ × 10^–5^*R*_GdL(exp)_		*a*	*b*	|*b*/*a*|
F^–^	1.39 ± 0.11	0.12	–0.7641	2.088	2.7
H_2_O	1.56 ± 0.12	0.51	–0.6740	1.955	2.9
O_ether_	2.06 ± 0.18	1.90	*-0.6525*	*1.861*	2.9
O_carboxyl_	2.14 ± 0.13	1.78	–0.6154	1.769	2.9
O_carbonat_	2.31 ± 0.12	1.58	*-0.5582*	*1.642*	2.9
N_amin_	3.67 ± 0.23	3.70	–0.3358	1.120	3.3
N_arom_	*3.11*		–0.4349	1.395	3.2

The parameters presented in Table 9 were obtained from the linear regression of the spectral data of the Gd(III) compounds listed in Table 7. The obtained β_i_ parameters are correlated with the Jørgensen’s h-nephelauxetic ones for coordinating atoms that are common to both studies.^46^ The order of the estimated nephelauxetic parameters in principle follows the ratio , where α is a ligand polarizability,^63^*Q* is a Bader charge, and Δχ is a Pauling electronegativity difference of Gd and donor atom. These results may suggest an increase of covalency in the Gd(III)–L bonds as follows: F^–^ < O_water_ < O_ether_ < O_carbox_ < O_carbonate_ < N_amino_. This sequence is, in principle, in good agreement with the order of the experimental bond degrees presented in Table 4. To support this conclusion, it is worth considering the experimental electron density in bond critical point ρ_c_ as a function of Gd(III)–L bond lengths. In general, the ρ_c_ values are proportional to *R*^–*n*^. Because the most common Gd–L bond lengths vary within a relatively narrow range^64^ (Figure 4) and because it is possible to distinguish the most characteristic bond length range for the each type of bond, the function ρ_c_ = *f*(*R*_GdL_) was assumed to be linear (eq 3).
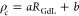
3

**Figure 4:**
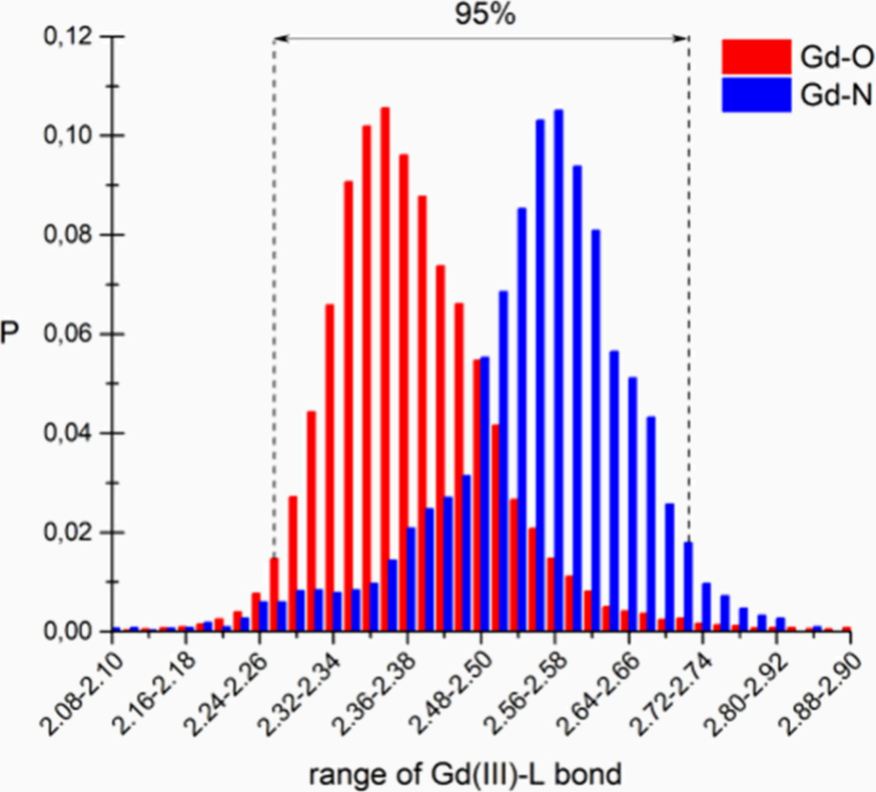
Variation of Gd–O and Gd–N bond lengths. *P* is the probability of the occurrence of a given Gd–L bond lenghts. The data were taken from Cambridge Crystallographic Data Centre.^64^

The determined values of the slope (*a*) and the intercept (*b*) characteristic for a given Gd–L bond are also presented in Table 9. The ρ_c_ values obtained for the other Gd(III) compounds, namely, [GdCl*(o*-phenanthroline)_2_(H_2_O)_3_]Cl_2_(H_2_O)^52^ and [Gd(H_2_O)_9_](CF_3_SO_3_)_3_,^49^ have also been taken into account in the calculation (Table 9).

Inspection of Table 9 reveals that the nephelauxetic β parameters follow the order observed for the slope *a* and the intercept *b* in eq 3; they are highly correlated, and the Pearson correlation coefficient was found to be *P*(β, *a*) = 0.9903, *P*(β, *b*) = −0.9978, *P*(*a*, *b*) = −0.9972. This allows one to determine β as a function of *a* or *b* (eqs 4a–4c).
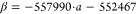
4a
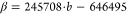
4b
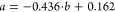
4cBased on these relationships, the *a* and *b* values for the other O_ether_ and CO_3_^2–^ ligands as well as the N_arom_ β values were interpolated (Table 9).

In general, the shorter the Gd(III)–L bonds are, the larger the ρ_c_ values. The slope |*a*| values are larger for more electronegative donor atoms; in other words, for more electronegative donor atoms, the ρ_c_ value decreases more rapidly on increasing the Gd(III)–L distance (Figure 5). Thus, it is possible to indicate a Gd(III)–L bond length (∼2.5 Å) above which the ρ_c_ value is larger for less electronegative donor ligands than for more electronegative ones. The |*b*/*a*| value may be, in turn, an indicator of the Gd(III)–L distance at which the ρ_c_ value is practically equal to zero. This does not mean, however, that there is no Gd(III)–L interaction but rather that the interaction can be treated as essentially electrostatic. Taking into account all the above-mentioned features and correlations between ρ_c_, *a*, *b*, and the nephelauxetic β parameter, it is likely that the β parameter may reflect penetration degree of the electron lone pairs of the ligands inside the metal basin (Gd(III)). It has been found that the β parameter is proportional to the ratio , as was mentioned above. To verify this conclusion we have checked out how the nephelauxetic *h* parameters derived for transition metal complexes for the other ligands^46^ depend on the  ratio (Table S3). Indeed, it turned out that the correlation coefficient between both quantities was found to be 0.97.

**Figure 5:**
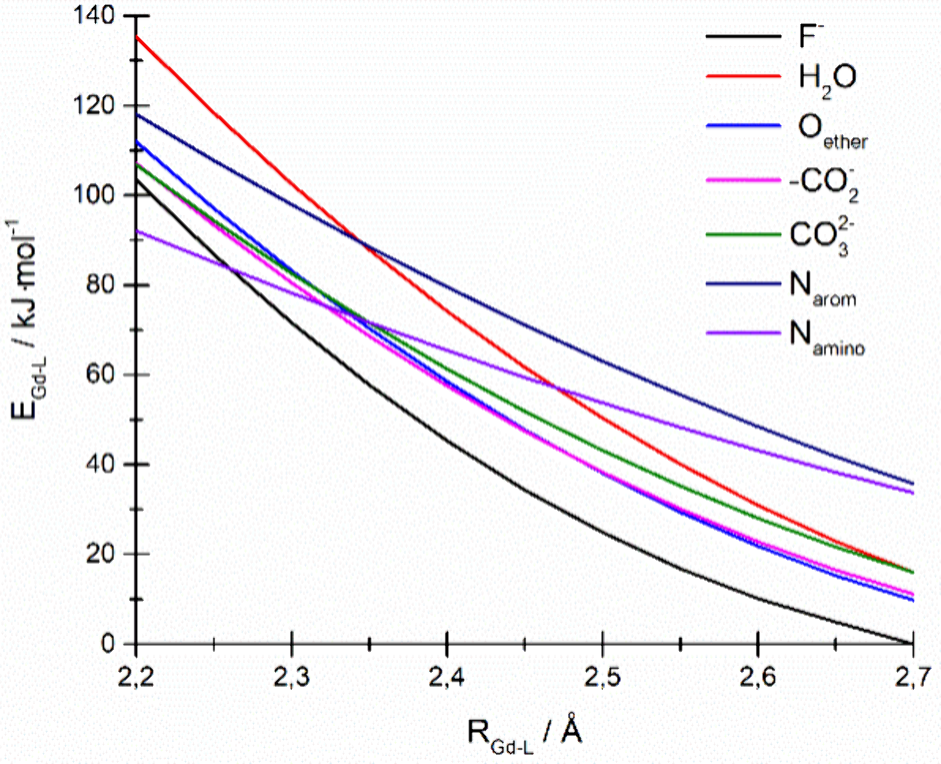
Plot of the bond energy vs Gd–L bond length.

The problem of Gd(III)–L bond energy is another issue that seems to be directly related to covalency and the spectroscopic parameters mentioned above. According to the relation suggested by Espinosa,^48^ the bond energy may be estimated from the density of potential energy according to the relation
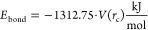
5It has been found that the density of the potential energy *V*(*r*_c_) for the studied systems may be fitted as an empirical square function of the electron density at the bond critical point ρ_c_ (Table S4) and accordingly the bond energy may be expressed as
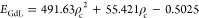
6aor
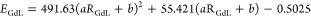
6bThe plot of the bond energy versus Gd–L distances for a given type of bond is shown in Figure 5.

The range of the Gd(III)–L bond lengths between 2.2 and 2.7 Å presented in Figure 5 has been reported as the most common for the various Gd(III) coordination compounds (Figure 4). The energy of the Gd(III)–L bonds depends on the chemical properties of the donor atoms and decreases more rapidly with the growth of the bond length for more electronegative donor atoms. Substantial diversity of Gd(III)–L bond lengths results in the energy of bonds may be the same or similar despite the considered Gd(III)–L bond lengths differing and vice versa, the energy of bonds may be different for similar bond lengths. Finally, the question arises whether the sum of the Gd(III)–L bond energy (∑*E*_GdL_) is correlated with the position of gravity center of an ^8^S_7/2_ → ^2*S*+1^L_J_ transition. As may be seen in Figure 6, there is strong positive correlation between both quantities, with the Pearson coefficient *P*(∑*E*_GdL_, *v̅*) being 0.97 and 0.92 for 8- and 9-coordinate species, respectively, and two well-defined linear relationships for both coordination numbers can be set up. In general, this correlation may suggest that an increase of bathochromic shift of the f–f bands is associated with a decrease in the Gd(III)–L bond energy.

**Figure 6:**
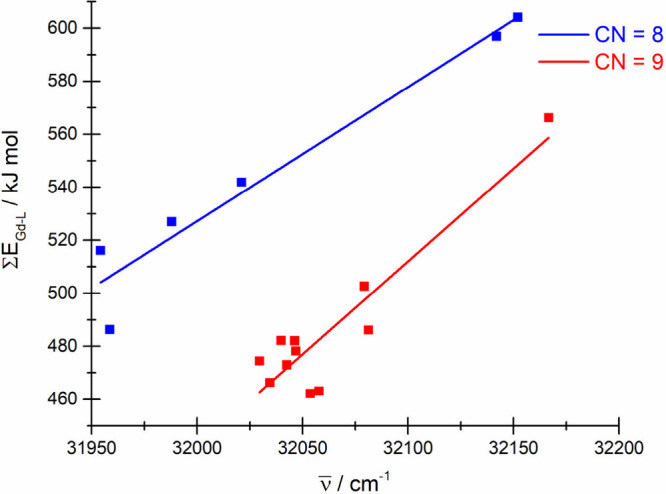
Plot of the sum of Gd–L bond energy versus energy of the gravity center of the ^6^P_7/2_ level.

Next, the Gd–OH_2_ bond energy difference between the corresponding two variously hydrated species (eqs 7 and 8) was estimated. The energies of the respective Gd(III) species were calculated as the sums of the contributions of the Gd(III)–L bond energies taken from eq 6 and the parameters listed in Table 9.
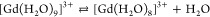
7
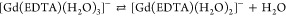
8Accordingly, the energy differences between the dehydrated and hydrated entities were found to be 35 kJ/mol for the [Gd(H_2_O)_*n*_]^3+^and 45 kJ/mol in the case of [Gd(EDTA)(H_2_O)_*n*_]^−^ complexes. The released water molecule forms new hydrogen bonds with 1.5 water molecules on average,^65^ and the enthalpy of this process is negative and typically varies in the range of −16 to −60 kJ/mol.^66,67^ For this reason the enthalpy values of reactions 7 and 8 in solution should be equal to the Gd–OH_2_ bond energy plus the hydrogen bond formation enthalpy. Indeed, the experimental values of Δ*H* were found to be −6 ± 2 kJ/mol and 18 ± 3 kJ/mol for reactions 7 and 8, respectively,^38^ and it may be noticed that the calculated enthalpy of the dehydration reactions in solution agrees well the experimental values.

The intensity Judd–Ofelt Ω_λ_ parameters^68,69^ for the selected crystals were also determined from the same set of transitions. It should be noted that the Ω_4_ parameter was omitted from the calculations because out of the transitions taken into account only the ^8^S_7/2_ → ^6^D_9/2_ one has a small, nonzero U^4^ value. The determined Ω_2_ and Ω_6_ parameter values are presented in Table 10.

**Table 10:** Judd–Ofelt Ω_2_ and Ω_6_ Parameters as Well as Experimental and Theoretical Luminescence Lifetimes for the Crystals under Study

	Compound	Ω_2_ × 10^20^ cm^2^	Ω_6_ × 10^20^ cm^2^	τ_exp_/τ_calc_ μs
1	[Gd(H_2_O)_8_]^3+^	1.58 ± 0.39	3.37 ± 0.05	8.26/8.95
3	[Gd(H_2_O)_9_]^3+^	0.44 ± 0.93	2.60 ± 0.13	14.9/13.0
4	[Gd(CO_3_)_4_(H_2_O)]^5–^	5.54 ± 0.94	2.91 ± 0.08	5.62/6.66
5	[Y:Gd(CO_3_)_4_]^5–^	17.2 ± 0.61	4.17 ± 0.09	3.45/3.55
6	[Gd(EDTA)(H_2_O)_3_]^−^	3.56 ± 1.11	5.84 ± 0.16	8.59/7.36
9	[Gd(EDTA)F_2_]^3–^	1.77 ± 0.81	4.42 ± 0.09	6.14/6.55
10	[Gd(CDTA)CO_3_]^3–^	3.94 ± 0.90	5.94 ± 0.12	7.39/6.01
11	[Gd(HDTPA)(H_2_O)]^2–^	3.07 ± 0.37	6.09 ± 0.05	3.37/8.48
12	[Gd(DTPA)(H_2_O)]^2–^	2.36 ± 0.56	6.69 ± 0.08	6.35/7.84
13	[Gd(DTPA)F]^3–^	4.39 ± 0.38	6.62 ± 0.05	5.26/7.84
14	[Gd(EGTA)(H_2_O)]^−^	4.35 ± 0.73	5.97 ± 0.10	7.20/7.51
17	[Gd(NTA)_2_(H_2_O)]^3–^	1.74 ± 1.69	5.50 ± 0.13	8.00/9.73
18	[Gd(DOTA)(H_2_O)]^−^	2.45 ± 1.20	4.51 ± 0.17	8.85/9.72

The oscillator strength of the ^8^S_7/2_ → ^6^P_7/2_ band enabled us to determine radiation probabilities as well as the luminescence lifetime of the ^6^P_7/2_ state. The results are also collected in Table 10.

As it may be seen, the Ω_2_ parameter values change to a greater extent (from 0.44 ± 0.93 up to 17.2 ± 0.61) in comparison with Ω_6_. In general, the Ω_2_ parameter is particularly sensitive to changes in the chemical properties of the donor atom ligands and the geometry of the first coordination sphere. In the case of Gd(III) spectra, however, no transitions are characterized by a large value of the reduced matrix element of the unit tensor operator U^(2)^; therefore, none of the bands observed in UV may be regarded as hypersensitive. Thus, the interpretation of the Ω_2_ parameter changes is more difficult. The results indicate that the Ω_6_ parameter exhibits a moderate correlation with the estimated values of the Gd–L bond energy, with a negative Pearson coefficient of −0.69. Due to the increased influx of electron density to the 6s orbitals, which leads to the screening and the contraction of the 5d shell, the reduction in the ⟨4f|*r*^*n*^|5d⟩ radial integrals, to which the Ω_6_ parameter is particularly sensitive, takes place.^70,71^ These results are consistent with those obtained for the Ln(III) tetracarbonate systems, published by us previously.^20^ The calculated and experimental luminescence lifetimes of excited ^6^P_7/2_ are similar within experimental error, confirming thus that the luminescence quantum yield of the Gd(III) compounds is close to 100%. Inspection of Table 10 reveals that for the same type of compound (1, 2, 3 and 4) the decrease of the coordination number brings about shortening of the luminescence lifetime. This feature seems to be also connected with covalency effects.

In conclusion, the crystal structures of the following compounds were determined: [C(NH_2_)_3_]_3_[Gd(EDTA)F_2_]·H_2_O, [C(NH_2_)_3_]_3_[Gd(DTPA)F]·H_2_O, [C(NH_2_)_3_]_3_[Gd(CDTA)(CO_3_)]·H_2_O, and Na_2_[Gd(DTPA)(H_2_O)]·7.875H_2_O. Out of these compounds, the [C(NH_2_)_3_]_3_[Gd(EDTA)F_2_]·H_2_O crystals turned out to be the most suitable for the XREDD measurements.

The coordination sphere of the Gd(III) cation in this complex is occupied by four oxygen atoms, two nitrogen atoms, and two fluorine atoms. The Gd–L bond lengths change in the order Gd–F (av, 2.24(2)) < Gd–O (av, 2.37(1)) < Gd–N (av, 2.65(19)). The determined values of the Laplacian of the electron density at the bond critical points, ∇^2^ρ_c_ > 0, and the bond degrees, BD < 0, enabled us to find that the Gd–O, Gd–N, and Gd–F bonds may be classified as transit closed shell ones, TCS, or as polarized covalent ones. The DFT calculations confirm these conclusions.

The natural charge of the Gd is 1.86, and the natural configuration of the cation is [Xe]6s^0.13^4f^7.10^5d^0.83^. In comparison with the [Gd(H_2_O)_9_](CF_3_SO_3_)_3_ crystal for which the Gd charge was found to be 1.96 and the configuration [Xe]6s^0.16^4f^7.10^5d^0.69^, in the present case the transfer of charge onto the metal is greater and it is directed predominantly to the 5d orbitals of Gd cation. Simultaneously, the donation of the charge onto 6s and 4f orbitals occurs to a lesser extent. The compounds with lower coordination number are characterized by larger donation of the ligand charges onto the Gd(III) in comparison with higher coordinated complexes.

The experimental values of the Bader charges of the fluorine anions were found to be −0.58 and −0.72, and interestingly, their charges are less negative than those of the carboxylate O atoms (−1.15 to −1.25). Furthermore, the charges of the nitrogen atoms are −0.84 and −0.85. It was observed that in a narrow range of the Gd–L bond lengths, the ρ_c_ and *R*_GdL_ values are linearly dependent.

It has been shown experimentally that even for F-, O-, and N-donor ligands, the Gd(III)–L interaction should be classified as transition closed shell TCS interaction, and the covalent contribution to Gd(III)–L bonds may be more common than usually realized.

In general, the shorter the Gd(III)–L bond length is, the larger the ρ_c_ value. For more electronegative donor atoms, the ρ_c_ value decreases more rapidly on increasing the Gd(III)–L distance. From the linear fitting of ρ_c_ vs *R*_GdL_, the distance beyond which the Gd(III)–L interaction can be treated as essentially electrostatic has been estimated.

Next, 18 monocrystalline samples of Gd(III) compounds with organic and inorganic ligands (EDTA, CDTA, DTPA, NTA, EGTA, CO_3_^2–^, H_2_O, and F^–^) were characterized by UV absorption and emission spectroscopy. It was found that the gravity centers of the ^8^S_7/2_ → ^6^P_7/2_, ^6^P_5/2_, ^6^P_3/2_, ^6^I_7/2_,^6^D_9/2_ transitions depend on the type of the coordinated atoms and their distance from the central Gd(III) cation. The empirical relation connecting these quantities has been found. It has been shown that the partial nephelauxetic β_i_ parameters reflect the penetration degree of electron lone pairs of ligands inside the metal basin. The β_i_ parameters are strongly correlated with the *a* and *b* parameters from the linear fitting of ρ_c_ (ρ_c_ = *aR*_GdL_+ *b*) derived from the XREDD experiments.

Subsequently, the bond energy was estimated from the density of potential energy derived from the XREDD experiment, and it was found to depend on the chemical properties of the donor atoms and to decrease with increasing bond length; the decrease was more rapid for more electronegative atoms. Finally, it was found that the sum of the Gd(III)–L bond energy is correlated with the position of gravity center of the ^8^S_7/2_ → ^2*S*+1^L_J_ transitions. In general, these correlations suggest that increase of covalency in the Gd–L bonds is associated with a decrease in their bond energy; namely, because of the charge transfer from a ligand to the Gd(III) cation, the ionic attraction between them decreases.

The nature of the Gd(III)–L bond character is also reflected in the thermodynamic properties of the Gd(III) compounds. The estimated enthalpy of the dehydration reaction is in good agreement with that determined experimentally. We have also shown that an increase of a covalency in Gd(III)–L bonds brings about decrease of the Ω_6_ intensity parameter and luminescence lifetimes of the ^6^P_7/2_ state.

## Experimental Methods

*Sample Preparation*. The crystals of compounds under study were obtained according to the procedure described elsewhere.^20,38^ Aqueous suspension of Gd_2_O_3_ and an appropriate solid polyaminopoly(carboxylic acid)(PAC) was heated at 90 ± 5 °C for about 24 h. Next, solid [C(NH_2_)_3_]_2_CO_3_ or NaOH/KOH was added to dissolve the white precipitate. The amounts of the respective compounds used for the preparation of the crystals are given in Table 11.

**Table 11:** Amounts of Compounds Used for the Preparation of Crystals under Study^a^

Crystal	Gd_2_O_3_ [g]	PAC [g]	[C(NH_2_)_3_]_2_CO_3_ [g]	MOH [g]	HF [ml]
[C(NH_2_)_3_]_3_[Gd(EDTA)F_2_]·H_2_O	0.373	0.614	1.111	–	0.36
[C(NH_2_)_3_]_3_[Gd(DTPA)F]·H_2_O	0.381	0.780	1.135	–	0.37
[C(NH_2_)_3_]_3_[Gd(CDTA)(CO_3_)]·H_2_O	0.370	0.818	4.042	–	–
Na_3_[(DTPA)(H_2_O)]·7.875H_2_O	0.366	0.810	–	NaOH 0.165	–
K_3_[Gd(NTA)_2_(H_2_O)]·5.5H_2_O*	0.725	1.552	–	KOH 0.69	–
Na[Gd(EDTA)(H_2_O)_3_]·5H_2_O*	1.002	1.620	–	NaOH 0.22	–

a The crystals were checked by X-ray diffraction, and they were isomorphic with those published previously.^72,73^

*Preparation of the [C(NH_2_)_3_]_5_[Y:Gd(CO_3_)_4_]·2H_2_O Crystals*. To an aqueous solution (20 mL) containing 50 mmol of [C(NH_2_)_3_]_2_CO_3_ (ABCR) was added an aqueous solution (10 mL) of YCl_3_ (125 mmol) and 0.50 mL of GdCl_3_ (0.050 mmol). The solution was heated at ∼90 °C under reflux until the initially formed precipitates dissolved. The final pH of the solution was approximately 12. Large (5 mm diameter) crystals of the formula [C(NH_2_)_3_]_5_[Y_0.967_Gd_0.033_(CO_3_)_4_]·2H_2_O were formed during slow evaporation of the solvent. The concentrations of Y(III) and Gd(III) in the crystals were determined by inductively coupled plasma atomic emission spectroscopy using a spectrometer (ARL Model 3410 ICP OES). The obtained crystals were isostructural with those previously published.^20^

*Crystal Structure Determination*. Suitable crystals were cut from larger ones, mounted on a Rigaku diffractometer equipped with a CCD counter, and measured at 100 K. The structures were solved routinely by using Patterson synthesis. The C- and N-bonded hydrogen atoms were placed in positions calculated from the geometry, and those bonded to O atoms were found from difference Fourier maps; not all were found. The final refinements were anisotropic for all non-H atoms. The computations were performed with the SHELXS98^74^ and SHELXL^75^ programs, and the molecular graphics was prepared with DIAMOND.^76^ The details of the data collection and the structure refinement of the crystals under study are given in Table S1 in the Supporting Information.

[C(NH_2_)_3_]_3_[Gd(EDTA)F_2_]·H_2_O, CCDC 2363075; [C(NH_2_)_3_]_3_[Gd(DTPA)F]·H_2_O, CCDC 2363074; [C(NH_2_)_3_]_3_[Gd(CDTA)CO_3_]·H_2_O, CCDC 2363076; and Na_2_[Gd(DTPA)(H_2_O)]·7.875H_2_O, CCDC 2363077.

*X-ray Data Collection and Multipole Refinement of [C(NH_2_)_3_]_3_[Gd(EDTA)F_2_]·H_2_O*. An appropriate crystal was cut from a larger one. The data were collected at 80 K up to 1.19 Å^–1^ (2θ_max_ = 115°) and then corrected for Lorentz and polarization factors as well as for absorption, the latter calculated from the crystal habit. The intensities were merged with *SORTAV.*(77) During the multipole refinements the positions of H atoms were refined with the C–H distances constrained to 1.09 Å, N–H to 1.01 Å, and O–W to 0.98 Å. The temperature factors were harmonically anisotropic for non-H atoms and isotropic for H ones. The multipole refinement was performed with the *XD2016* suite of programs^78^ against *F*^2^ up to 1.15 Å^–1^ using Hansen and Coppens formalism,^79^ in which the electron density is the sum of atomic contributions expressed as
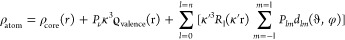
9where ρ_core_ and ρ_valence_ are the spherically symmetric electron-density functions of the core and valence electrons; *P*_v_ is the electron population parameter of the valence shell; κ is the contraction/expansion parameter of the valence shell radial electron-density function; κ′ and *P*_*lm*_ represent the contraction and population parameters of the multipole functions, respectively; *d*_*lm*_ are the spherical harmonic functions in real form; and *r*, ϑ, ϕ are the spherical coordinates. The atom scattering factors, ρ_core_ and ρ_valence_, were calculated using relativistic wave functions for neutral atoms, calculated at the PBE/QZ4P level of theory (A. Volkov and P. Macchi, xd.bnk_PBE-QZ4P-ZORA, unpublished), except for Gd, for which relativistic functions of Gd(III) (together with the virtual 5d and 6s ones) were calculated (using PBE^80^ functional, basis QZ4P, ZORA,^81^ noncollinear spin-orbit coupling; the calculations were performed with *ADF2019*(82)). The radial functions *R*_*l*_ were the Slater functions in the form
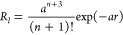
10where *n* may be different for each multipole order. The *n* values were standard, except for Gd, which expanded up to hexadecapoles (*n* = 9, 6, 6, 6, 6). For Gd, two monopoles, the valence and the deformation ones, were refined. Other non-H atoms were expanded up to octapoles and the H atoms up to quadrupoles. During refinement the multipoles of the F atoms tended to contract abnormally; therefore, their κ′ parameters were fixed at 1.4. Correction for isotropic extinction (mosaic distribution dominated, Gaussian distribution) was included. All non-H atoms were refined with harmonic anisotropic thermal vibration factors; N- and O-bonded H atoms were isotropic, and anisotropic harmonic thermal vibration factors for C-bonded H atoms were calculated with the SHADE3 server.^83^ The details of data collection and basic refinement information are presented in Table S1, and other details may be found in the Supporting Information.

*Theoretical Calculations*. The DFT calculations were performed with the ADF2019 suite of programs, using PBE functional and Slater TZ2P basis functions (TZ2P+ for Gd),^84^ and relativistic effects were included with a scalar ZORA approach. The calculations were performed for two systems: (a) a cluster {[Gd(EDTA)F_2_][C(NH_2_)_3_]_4_}^+^, devised so as to fence the atomic basins of the fluoride anions, to gain the Bader charges, the topological parameters, and quantities derived from them; (b) the complex anion [Gd(EDTA)F_2_]^3–^, to analyze its electronic structure in terms of the NBO^85−87^ approach. The crystal environment was simulated with *COSMO*(88,89) by placing the cluster or the complex anion, respectively, in a cavity of dielectric medium simulating acetic acid. The kinetic (*G*), potential (*V*), and total (*E*) energy densities, as well as the bond degrees (BD), both experimental and theoretical, were calculated according to the literature (eqs 11^90^, 12(91), and 14(48)):
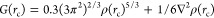
11
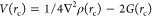
12
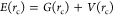
13
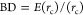
14where ρ is the electron density and *r*_c_ is the position of the bond critical point.

*UV–Vis Electronic Spectroscopy*. The ultraviolet electronic absorption spectra of the crystals under study were recorded with a Cary 5000 spectrophotometer at room temperature. Luminescence spectra and lifetimes of the studied crystals were measured with an Edinburgh Instruments FLS 920 spectrometer.

The experimental values of the oscillator strengths (*P*_exp_) were determined by means of eq 15:
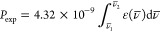
15ε is the molar absorption coefficient as a function of the wavenumber (***ν*****®**) in cm^–1^. The experimental *P*_exp_ values were used for the calculation of phenomenological Judd–Ofelt Ω_λ_ intensity parameters^68,69^ according to eq 16
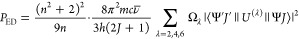
16*P*_ED_ is the oscillator strength of the electric dipole transition; *n* is the refractive index (*n* = 1.5); *m* is the electron mass; *c* is the speed of light; *v̅* is the wavenumber of the band maximum in cm^–1^; *h* is Planck’s constant; and *J* is the ground state quantum number.

The reduced matrix elements of the respective unit tensor operator *U*^(λ)^ are given by ⟨Ψ′*J*′∥*U*^(λ)^∥Ψ*J*⟩. The values of ⟨Ψ′*J*′∥*U*^(λ)^∥Ψ*J*⟩^2^ = U^(λ)^ operator were obtained from the paper by Carnall et al.^92^

The accuracy of the intensity parameter fitting was determined by the root-mean-square deviation (RMS), as defined by the following equation:
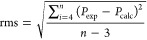
17where *P*_calc_ is the oscillator strength calculated from eq 16 and *n* is the number of absorption bands.

The calculated luminescence lifetimes from the absorption spectrum of the ^8^S_7/2_ → ^6^P_7/2_ were determined according to eq 18.^93,94^
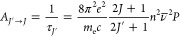
18where *A*_*J*′→*J*_ is radiative probability of the *J*′ → *J* transition, *τ*_*J*′_ the luminescence lifetime, *e* the electron charge, *m*_e_ the mass of electron, *c* the speed of light, *ν̅* the wavenumber of the band maximum, *n* the refractive index (1.5), and *P* the respective oscillator strength.

## Supporting Information Available

Residual density map, the section is through the plane defined by O1, Gd and F1 (Figure S1); selected structural data of crystals under study (Table S1); gravity center of the excited states and their crystal field splitting in the UV spectra of Gd(III) compounds (Table S2); nephelauxetic, ligand polarizability, charge, and optical electronegativity parameters (Table S3); structural and XREDD parameters for Gd(III) compounds (Table S4) (PDF)
GdEDTAF2 jz4c01903_si_002 Crystallographic information file (CIF)
GdCDTACO3 jz4c01903_si_003 Crystallographic information file (CIF)
NaGdDTPAH2Ojz4c01903_si_004 Crystallographic information file (CIF)
GdDTPAF jz4c01903_si_005 Crystallographic information file (CIF)

## Supplementary Material

jz4c01903_si_006.pdf

jz4c01903_si_002.cif

jz4c01903_si_003.cif

jz4c01903_si_004.cif

jz4c01903_si_005.cif
