# Supplementary material for: In Search of Covalency Measure of Gd(III)-Ligand Interactions
Source: J Phys Chem Lett. 2024 Sep 17;15(38):9723–37. doi: 10.1021/acs.jpclett.4c01903 (PMC11440599; doi:10.1021/acs.jpclett.4c01903)
Supplement: Supplementary file 1 — jz4c01903_si_006.pdf [file jz4c01903_si_006.pdf]

In search of covalency measure of Gd(III)-ligand interactions

Rafał Janicki, Miłosz Siczek and Przemysław Starynowicz*

AUTHOR INFORMATION

University of Wrocław, Faculty of Chemistry, F. Joliot Curie 14, 50-383 Wrocław

Corresponding Author

*Rafał Janicki: rafal.janicki@uwr.edu.pl

ElectronicSupplementary Information

**Figure S1.**	Residual density map, the section is through the plane defined by O1, Gd and F1………………………………………	2
**Table S1.**	Selected structural data of crystals under study…………………………………………………………………………	3
**Table S2.**	Gravity centre of the excited states and their crystal field splitting in the UV spectra of Gd(III) compounds……………………………………………………………………………………………………	4
**Table S3.**	Nephelauxetic h, ligand polarizability - α, charge - Q, optical electronegativity - χ parameters……………………………………………………………………………………………………	5
**Table S4.**	Structural and XREDD parameters for Gd(III) compounds……………………………………………………	6

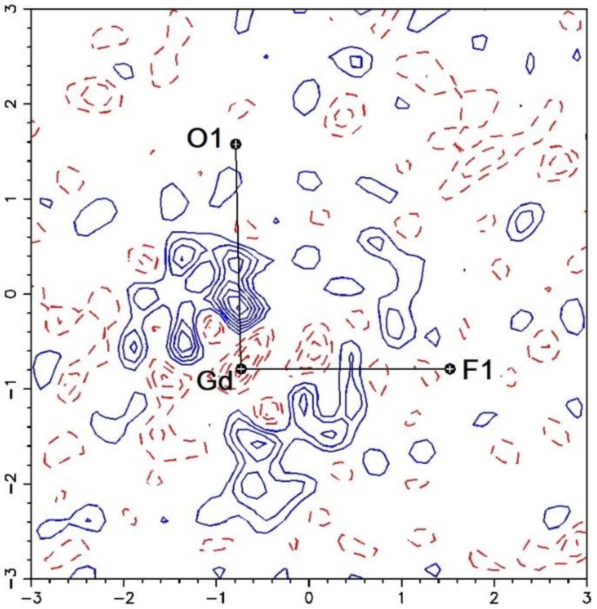

FigureS1. Residual density map, the section is through the plane defined by O1, Gd and F1. The contours (positive values – solid, blue; negative ones –dashed, red) are drawn every 0.1 e A ° _3. The zero contour has been omitted.

Table S1. Selected structural data of crystals under study

Crystal data				
CCDC	2363075	2363076	2363074	2363077
Chemicalformula	$C_{13}H_{32}F_2GdN_{11}O_9$	$C_{15}H_{18}GdN_2O_{11}·3(CH_6N_3)·2(H_2O)$	$C_{14}H_{18}FGdN_3O_{10}·3(CH_6N_3)·8(H_2O)$	$C_{14}H_{33.75}GdN_3Na_2O_{17.875}$
M_r	681.74	775.86	888.95	733.42
Crystal system, spacegroup	Monoclinic, $P2_1/n$	Monoclinic, $P2_1/c$	Triclinic, $P1$	Monoclinic, $P2_1/c$
Temperature (K)	80	100	100	100
a, b, c (Å)	13.475 (2), 10.435 (2), 17.432 (6)	17.804 (4), 15.513 (3), 10.659 (3)	10.180 (2), 12.946 (3), 13.766 (3)	22.488 (2), 17.886 (2), 26.482 (3)
β (°)	100.50 (2)	93.07 (3)	76.86 (3), 83.57 (3), 89.36 (3)	105.37 (1)
V (Å^3)	2410.1 (10)	2939.7 (12)	1755.4 (7)	10270.6 (19)
Z	4.0	4	2	16
Radiationtype	Mo $K\alpha$	Mo $K\alpha$	Mo $K\alpha$	Mo $K\alpha$
μ (mm^{-1})	2.83	2.34	1.98	2.70
Crystalsize (mm)	0.47 × 0.32 × 0.18	0.30 × 0.10 × 0.02	0.15 × 0.15 × 0.03	0.26 × 0.18 × 0.10
Data collection				
Diffractometer	Xcalibur, Ruby, Gemini ultra	XtaLABSynergy R, DW system, HyPix-Arc 150	XtaLABSynergy R, DW system, HyPix-Arc 150	XtaLABSynergy R, DW system, HyPix-Arc 150
T_{min}, T_{max}	0.378, 0.690	0.614, 1.000	0.655, 1.000	0.600, 0.830
No. of measured, independent and observed (?) reflections	166858, 33314, 30608	43666, 8930, 7681	29401, 10616, 9809	168614, 59730, 42781
R_{int}	0.020	0.038	0.032	0.039
(sin θ/λ)$_{max}$ (Å^{-1})	1.187	0.714	0.714	1.049
Refinement				
$R[F^2 > 2\sigma(F^2)]$, $wR(F^2)$, S	0.013, 0.037, 1.20	0.022, 0.050, 1.03	0.031, 0.081, 1.10	0.041, 0.101, 1.05
No. of reflections	27667	8930	10616	59730
No. of parameters	1240	413	458	1369
Δρ$_{max}$, Δρ$_{min}$ (e Å^{-3})	0.84, −0.59	1.08, −0.58	2.50, −1.83	3.65, −2.23

Table S2. Gravity centre of the excited states and their crystal field splitting in the UV spectra of Gd(III) compounds

Compound		$^6P_{7/2}$	$^6P_{5/2}$	$^6P_{3/2}$	$^6I_{7/2}$	$^6I_{9/2}$
$[Gd(H_2O)_8]Cl_3 \cdot C_{10}H_{20}O_5$	$\bar{\nu}_{GC}$	32142	32740	33314	35882	39609
	ΔCFS	74	51	17	67	116
$[Lu:Gd(H_2O)_8]Cl_3 \cdot C_{12}H_{24}O_6$	$\bar{\nu}_{GC}$	32152	32722	33309	35870	39629
	ΔCFS	84	28	-	85	96
$[Gd(H_2O)_9](CF_3SO_3)_3$	$\bar{\nu}_{GC}$	32167	32760	33342	35908	39683
	ΔCFS	29	28	0	24	19
$[C(NH_2)_3]_5[Gd(CO_3)_4(H_2O)] \cdot 2H_2O$	$\bar{\nu}_{GC}$	32030	32619	33195	35772	39502
	ΔCFS	128	112	44	64	109
$[C(NH_2)_3]_5[Y:Gd(CO_3)_4] \cdot 2H_2O$	$\bar{\nu}_{GC}$	31954	32571	33134	35715	39414
	ΔCFS	89	16	-	68	125
$[C(NH_2)_3][Gd(EDTA)(H_2O)_3]$	$\bar{\nu}_{GC}$	32081	32672	33252	35823	39552
	ΔCFS	88	66	32	52	84
$Na[Gd(EDTA)(H_2O)_3]\cdot H_2O$	$\bar{\nu}_{GC}$	32079	32669	3325-	35819	39555
	ΔCFS	97	98	37	55	69
$[C(NH_2)_3]_2[Lu:Gd(EDTA)(H_2O)_2]ClO_4 \cdot 4H_2O$	$\bar{\nu}_{GC}$	31988	32572	33124	35746	-
	ΔCFS	26	48	-	105	-
$[C(NH_2)_3][Gd(EDTA)F_2]\cdot H_2O$	$\bar{\nu}_{GC}$	32021	32614	33196	35760	39455
	ΔCFS	129	83	0	129	184
$[C(NH_2)_3]_3[Gd(CDTA)CO_3]\cdot H_2O$	$\bar{\nu}_{GC}$	31959	32546	33124	35695	39389
	ΔCFS	88	70	26	110	180
$[C(NH_2)_2(N_2H_4)][Gd(HDTPA)(H_2O)]\cdot 2H_2O$	$\bar{\nu}_{GC}$	32047	32638	33214	35784	39493
	ΔCFS	104	83	39	68	52
$Na[Gd(DTPA)(H_2O)]\cdot 7.875H_2O$	$\bar{\nu}_{GC}$	32054	32634	33210	35787	39501
	ΔCFS	97	79	39	60	97
$[C(NH_2)_3]_3[Gd(DTPA)F]\cdot H_2O$	$\bar{\nu}_{GC}$	32040	32630	33201	35777	39494
	ΔCFS	96	91	44	64	109
$[C(NH_2)_3][Gd(EGTA)(H_2O)]\cdot 2H_2O$	$\bar{\nu}_{GC}$	32043	32635	33203	35778	39491
	ΔCFS	86	78	28	67	109
$Na[Gd(DOTA)(H_2O)]\cdot 4H_2O$	$\bar{\nu}_{GC}$	32035	32625	33204	35778	39491
	ΔCFS	123	86	37	54	103
$K[Lu:Gd(DOTA)] \cdot KCl \cdot 4.6H_2O$	$\bar{\nu}_{GC}$	31996	32579	-	-	-
	ΔCFS	102	-	-	-	-
$K_3[Gd(NTA)_2(H_2O)]$	$\bar{\nu}_{GC}$	32058	32653	33234	35779	39497
	ΔCFS	64	56	0	41	84
$Na_3[Gd(ODA)_3]\cdot 2NaClO_4\cdot 6H_2O$	$\bar{\nu}_{GC}$	32046	32643	33209	35781	39509
	ΔCFS	21	23	8	77	115

Table S3. Nephelauxetic h, ligand polarizability - α, charge - Q, optical electronegativity - χ parameters

	h	α	Q	χ	$\frac{\alpha \cdot Q}{\chi}$
S^{2-}	3.62	3.77	2	2.58	2.93
I^-	2.87	5.80	1	2.66	2.18
O^{2-}	1.67	1.41	2	3.44	0.82
F	0.80	0.68	1	3.98	0.17
H_2O	1.00	1.41	1.2	3.44	0.49
Cl^-	2.00	2.70	1	3.16	0.86
Br^-	2.30	3.40	1	2.96	1.15
CN^-	2.00	2.63	1	2.55	1.03

Table S4. Structural and XREDD parameters for Gd(III) compounds

Compound	Bond	RGd-L	ρ_c	-0.5Vr	Ref.
$[C(NH_2)_3]_3[Gd(EDTA)F_2]\cdot H_2O$	Gd—F1	2.2553	0.365	86.64	This paper
	Gd—F2	2.2252	0.388	94.52	This paper
	Gd—O1	2.367	0.311	65.64	This paper
	Gd—O3	2.3722	0.309	64.32	This paper
	Gd—O5	2.361	0.317	66.95	This paper
	Gd—O7	2.3828	0.303	63.01	This paper
	Gd—N1	2.6654	0.225	36.76	This paper
	Gd—N2	2.6386	0.234	39.38	This paper
$[Gd(H_2O)_9](CF_3SO_3)_3$	Gd-OH_2	2.4108	0.335	66.95	49
	Gd-OH_2	2.5208	0.255	44.63	49
[GdCl(*o*-phenanthroline)$_2(H_2O)_3]Cl_2(H_2O)$	Gd-OH_2 (O1)	2.3794	0.35	80.44	52
	Gd-OH_2 (O2)	2.378	0.35	79.92	52
	Gd-OH_2 (O3)	2.4113	0.33	72.04	52
	Gd-N(phenantr) (1')	2.5604	0.29	54.54	52
	Gd-N(phenantr) (10')	2.5524	0.29	55.79	52
	Gd-N(phenantr) (1)	2.5721	0.27	52.16	52
	Gd-N(phenantr) (10)	2.5456	0.28	54.61	52
